# Lifelong Effects of Thermal Challenges During Development in Birds and Mammals

**DOI:** 10.3389/fphys.2020.00419

**Published:** 2020-05-25

**Authors:** Andreas Nord, Sylvain Giroud

**Affiliations:** ^1^Section for Evolutionary Ecology, Department of Biology, Lund University, Lund, Sweden; ^2^Research Institute of Wildlife Ecology, Department of Interdisciplinary Life Sciences, University of Veterinary Medicine Vienna, Vienna, Austria

**Keywords:** body temperature, climate change, development, endotherm, heterothermy, phenotypic flexibility, temperature fluctuation, thermal adaptation

## Abstract

Before they develop competent endothermy, mammals and birds are sensitive to fluctuating temperature. It follows that early life thermal environment can trigger changes to the ontogeny of thermoregulatory control. At the ecological level, we have incomplete knowledge of how such responses affect temperature tolerance later in life. In some cases, changes to pre- and postnatal temperature prime an organism’s capacity to meet a corresponding thermal environment in adulthood. However, in other cases, developmental temperature seems to constrain temperature tolerance later in life. The timing, duration, and severity of a thermal challenge will determine whether its impact is ameliorating or constraining. However, the effects influencing the transition between these states remain poorly understood, particularly in mammals and during the postnatal period. As climate change is predicted to bring more frequent spells of extreme temperature, it is relevant to ask under which circumstances developmental thermal conditions predispose or constrain animals’ capacity to deal with temperature variation. Increasingly stochastic weather also implies increasingly decoupled early- and late-life thermal environments. Hence, there is a pressing need to understand better how developmental temperature impacts thermoregulatory responses to matched and mismatched thermal challenges in subsequent life stages. Here, we summarize studies on how the thermal environment before, and shortly after, birth affects the ontogeny of thermoregulation in birds and mammals, and outline how this might carry over to temperature tolerance in adulthood. We also identify key points that need addressing to understand how effects of temperature variation during development may facilitate or constrain thermal adaptation over a lifetime.

## Introduction

Mammals and birds are endotherms and, as such, control core body temperature (*T*_b_) by means of endogenous heat production across a vast temperature span. However, for the duration of embryonic development, and at least until adequate insulation has been attained, these animals are effectively poikilothermic, i.e., have limited ability to maintain *T*_b_ when ambient temperature (*T*_a_) fluctuates (e.g., Pereyra and Morton, 2001; Geiser et al., 2019). Hence, parents buffer changes in *T*_a_ to secure appropriate developmental conditions until offspring have attained endothermy. This is pivotal, because low *T*_b_ slows growth rate which may prolong both the embryonic period and time to independence with potential downstream ecological consequences (Remes and Martin, 2002; Cheng and Martin, 2012). Yet, because parents also need to self-feed and, in the case of altricial species, periodically leave the nest to provide for offspring, developing endotherms will be subjected to fluctuating *T*_a_, at least for some periods in some early life stages.

Given the sensitivity to perturbations when regulatory systems form (Burggren and Mueller, 2015; Eyck et al., 2019), the embryonic thermal environment can affect pre- and postnatal phenotypes. When there is substantial and sustained deviation from optimum developmental temperature, offspring may accrue congenital deficiencies (e.g., Lundy, 1969). Such pronounced challenges are arguably rare in nature. It may therefore be more relevant to consider effects of lower-intensity temperature variation, such as during unusually cold or warm breeding seasons, across a reproductive season, and in relation to variation in parents’ reproductive investment. This has been studied in some detail in poultry (e.g., Tzschentke and Nichelmann, 1999; Nichelmann and Tzschentke, 2002; Nichelmann, 2004). Broadly speaking, these efforts show that mild, short-duration, thermal stimuli before or shortly after hatching improve chicks’ capacity to deal with a corresponding challenge as juveniles and in adolescence. However, this work has unclear ecological relevance, because free-ranging animals are presumably adapted to more variable thermal environments, and face different thermoregulatory and energetic constrains, than poultry (e.g., Tickle et al., 2018; Tickle and Codd, 2019). Thus, work on wild birds indicates that mildly hypothermic incubation reduces, whereas mildly hyperthermic incubation increases, offspring cold tolerance (e.g., DuRant et al., 2012, 2013a,b). In contrast, short- and long-term effects of changes to rearing temperature on thermoregulation are poorly understood. In mammals, it is not known how offspring thermoregulation is affected by prenatal temperature, and data on rearing temperature-effects on thermal physiology in subsequent life stages are scarce.

**FIGURE 1:**
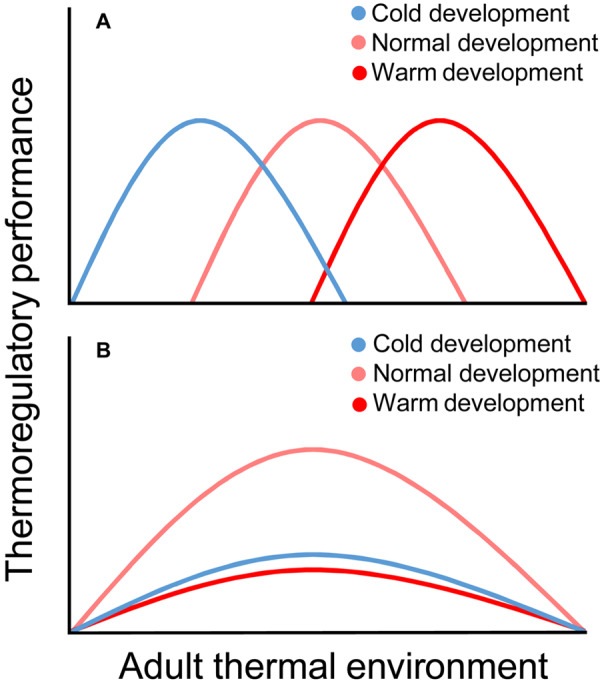
Possible effects of the thermal environment during development on thermoregulatory performance in adulthood. Under the environmental matching hypothesis **(A)**, adult performance is better when the thermal environment matches that in which the individual developed. Under the silver spoon hypothesis **(B)**, both colder-than-normal and warmer-than-normal developmental temperatures act suppressively on the pre- and/or postnatal phenotype, such that individuals that developed in unconstraining thermal regimes (here, “normal”) consistently perform better than cold- and warm-reared individuals as adults.

The lack of information on how developmental temperature affects adult thermoregulation is unfortunate, not the least considering the predicted increase of extreme temperature events (IPCC, 2013) that risks increasingly decoupling juvenile and adult thermal environments. It is conceivable that developmental-temperature-effects on adult thermoregulation can be broadly categorized as ameliorating or constraining (Figure 1): (a) if juveniles adapt non-reversibly to their thermal environment, then as adults we expect individuals to perform better in matched, and worse in mismatched, environments (“Environmental matching hypothesis”; Figure 1A); (b) if changes to developmental temperature constrain juvenile growth and maturation, we expect that individuals who were thermally challenged when growing up will consistently perform worse than those that developed in “normal” environments as adults (“Silver spoon hypothesis”; Figure 1B) (terminology after Monaghan, 2008). Here, we summarize the main findings for how developmental temperature affects the ontogeny of thermoregulation and how this links to adult thermoregulatory performance. We discuss the extent to which this may facilitate or constrain thermal adaptation in adulthood, and finish by addressing particularly pressing matters to investigate in this context.

## When and Why Does Developmental Temperature Vary?

### Mammals Before Parturition

Females of many mammals improve embryonic homeothermy by reducing circadian variation in *T*_b_ during gestation (e.g., Fewell, 1995; Trethowan et al., 2016; Wharfe et al., 2016; Thiel et al., 2019), and may even suppress febrile responses to protect the embryo from thermal damage (Begg et al., 2007) (but see Laburn et al., 1992). Even some heterotherms, which would normally display large daily or seasonal *T*_b_ reduction, are more homeothermic during pregnancy. For example, hibernating bears maintain stable *T*_b_ during gestation and only allow *T*_b_ to drop after parturition (Hissa, 1997; Tøien et al., 2011; Shimozuru et al., 2013; Friebe et al., 2014). Other heterotherms do use torpor when pregnant. This is typically a direct or preemptive response to energy shortage and is more common in species regularly facing energetic challenges during breeding, such as those reproducing when it is cold and those relying on ephemeral or seasonal/patchy forage (reviewed by Geiser, 1996; McAllan and Geiser, 2014). Females safeguarding energy balance in this manner do so at the expense of prolonged gestation (e.g., Racey, 1973) and possible phenotypic consequences to offspring resulting from a more variable developmental temperature.

### Mammals After Parturition

Until thermogenic capacity is sufficient for self-maintenance, mammals experience fluctuating *T*_b_ as determined by the amount of maternal brooding and thermal properties of the nest, and by the extent to which post-parturition females use torpor. Some of the fluctuations in nest temperature can be mitigated by huddling, which allows maintained growth rate even during a cold challenge (Gilbert et al., 2007, 2010, 2012). After weaning, young mammals are inevitably exposed to fluctuating *T*_a_ in line with habitat properties. Depending on reproductive period, juveniles of the same species might experience warm or cold temperatures during this time.

### Birds Before Hatching

Because birds have external development, embryos are more exposed to *T*_a_ compared to (non-monotreme) mammals. With some exceptions, such as the megapodes that utilize heat from decomposing material to incubate eggs (Booth and Jones, 2002) and some species that rely on solar incubation (e.g., De Marchi et al., 2008), heat for embryonic development is actively supplied by one or both parents. Some capital breeders, such as common eiders (*Somateria mollissima*), take no or few daily recesses from incubation (e.g., Kristjánsson and Jónsson, 2011). At the other extreme are single-sex intermittent incubators that leave the nest to forage several times per hour (Deeming, 2002). It follows that parental incubation behavior impacts the degree of temperature fluctuation experienced by the embryo. Moreover, incubation temperature is often lower in more strenuous conditions, such as in low *T*_a_ or during incubation of larger clutches (reviewed by Nord and Williams, 2015), because the energy costs of incubation constrain parental investment in keeping eggs warm (Williams, 1996; Tinbergen and Williams, 2002; Nord and Williams, 2015). Females of some species mitigate these costs by torpor (Calder and Booser, 1973; Kissner and Brigham, 1993), with inevitable consequences for embryonic temperature.

### Birds After Hatching

Once eggs hatch, chicks are brooded by one, or both, parents until thermogenic capacity and insulation are sufficient. Precocial species, that self-feed from hatching onward, are exposed to the elements during this time and will alternate short feeding bouts with being brooded by the parents (e.g., Pedersen and Steen, 1979). Altricial chicks are more strongly affected by *T*_a_, meaning *T*_b_ is influenced by the balance between parental provisioning and brooding. However, on account of the increase in thermal mass as chicks grow, the brood as a unit may be functionally homeothermic already a few days after hatching (Węgrzyn, 2013; Andreasson et al., 2016).

## Responses to Prenatal Temperature Variation

### Mammals

We are not aware of any studies that have tested how fluctuating temperatures *in utero* affect the subsequent thermoregulatory performance of juveniles and adults. This clearly needs further investigation (see section “Future Directions” below).

### Birds

The effects of embryonic temperature on postnatal thermoregulation have been studied particularly in poultry since temperature fluctuations inside rearing facilities have consequences for welfare and economic return (Naga Raja Kumari and Narendra Nath, 2018). Thermal sensitivity is the greatest when the hypothalamus-thyroid-pituitary-adrenal (HTPA) axis forms (Loyau et al., 2015), in line with the modulatory role of thyroid hormones in avian thermoregulation (Ruuskanen et al., 2019). In the chicken, this commences during the middle third of embryogenesis, when even brief (2–5 h) exposure to hypo- or hyperthermic incubation alters thyroid and glucocorticoid hormone secretion in response to a thermal challenge after hatching, and results in phenotypic changes that improve chicks’ capacity to deal with cold or heat at least until market age of ca. 35–50 days (e.g., Yahav et al., 2004; Shinder et al., 2009, 2011; Piestun et al., 2011). However, the effects appear to be different when the challenge is continuous. For example, periodic cooling during the entire incubation period in zebra finches (*Taeniopygia guttata*) increased embryonic metabolic rate, but decreased yolk conversion ratio, such that chicks hatched in poorer condition (Olson et al., 2006, 2008). Similarly, chickens incubated at constant low temperature produced less, not more, heat during acute cold exposure compared to controls (Black and Burggren, 2004). Moreover, Japanese quail (*Coturnix japonica*) chicks incubated in constant or cyclical low temperature were smaller, weighed less, and had elevated metabolic rate (after constant low incubation only) as adults relative to controls (Ben-Ezra and Burness, 2017).

Embryos of wild birds are adapted to the constantly fluctuating temperatures produced by parental behavior (above, and Webb, 1987). Yet, studies directly manipulating egg temperature in free-ranging birds largely corroborate findings in captive models. Accordingly, chronically low incubation temperature lowers body condition and elevates metabolic rate (Hepp et al., 2006; DuRant et al., 2011; Nord and Nilsson, 2011), and reduces the capacity to meet a cold challenge (DuRant et al., 2012, 2013a). None of these studies measured effects on thermoregulation once chicks were independent. Hence, it is unclear if incubation temperature-linked effects on survival in wild birds after fledging (Hepp and Kennamer, 2012; Nord and Nilsson, 2016; see also Berntsen and Bech, 2016) has a thermo-physiological basis.

It is not known if brief exposure to low incubation temperatures, similar to that in many poultry studies, affects offspring thermoregulation. This is unfortunate, because incubating birds sometimes prioritize self-maintenance by ceasing to incubate for several hours (e.g., MacDonald et al., 2013; reviewed by Nord and Williams, 2015). The resultant thermal challenge for embryos may be equivalent to when mammals enter torpor during gestation.

## Responses to Postnatal Temperature Variation

### Mammals

We are aware of only two studies reporting on how early life thermal conditions affect thermoregulation in adult mammals. In fat-tailed dunnarts (*Sminthopsis crassicaudata*), adults showed more frequent, deeper, torpor associated with significantly greater energy savings when they developed, and were subsequently kept, in cold compared to warm conditions (Riek and Geiser, 2012). In yellow-footed antechinus (*Antechinus flavipes*), rearing in warm conditions from weaning onward caused increased metabolic rate when adult females, but not males, were cold-exposed. After warm-exposure of adults that were reared in the cold, metabolic rate was significantly reduced for both sexes (Stawski and Geiser, 2020). Hence, developing in the warmth seems to reduce flexibility of the metabolic response to changing temperature, at least in males. In line with this, piglets exposed to heat stress during their first 10 days of life showed reduced thermo-tolerance when heat stressed at weaning compared to piglets reared in standard and cold conditions (Johnson et al., 2018).

Developmental temperature also affects morphology. In rats, warm-rearing from parturition increases the size and vascularity of thermolytic effectors (tail, salivary glands) (Demicka and Caputa, 1993a, b). While vascularity is likely amenable to subsequent thermal acclimation (e.g., Demicka and Caputa, 1993a), changes to external morphology (and associated heat transfer consequences) could remain over the animals’ lifespan.

### Birds

In the chicken, thermal manipulation for 12–24 h during the first week after hatching elicits responses largely analogous to those triggered by the same stimulus during incubation. Accordingly, heat- or cold-acclimation at this age improves control of *T*_b_ and survival when chicks are subsequently exposed to acute thermal stress at 6–7 weeks of age (Arjona et al., 1988, 1990; Yahav and Hurwitz, 1996; Shinder et al., 2002), possibly *via* acclimation of evaporative cooling capacity (Marder and Arieli, 1988; Midtgård, 1989). It is not clear if the causation is similar to that in the embryonic period. However, non-thermal challenges to young birds can bring lasting effects on glucocorticoid levels (e.g., Marasco et al., 2013), which suggest that the HTPA axis is still sensitive to developmental perturbations at this time.

In line with studies on mammals, postnatal *T*_a_ can affect the size of thermolytic effectors. Japanese quail reared in warm *T*_a_ developed smaller bills than birds reared in cold *T*_a_ (Burness et al., 2013). As adults, after nearly 3 months in common garden, warm-reared quail had higher bill temperature than cold-reared birds, particularly in low *T*_a_, indicating non-reversible changes to bill vasculature (Burness et al., 2013). It would be interesting to know if heart-weight reduction in warm-reared chickens (Yahav and Hurwitz, 1996), which has obvious links to circulation and thermoregulation, is equally non-reversible.

Only a handful of studies have manipulated rearing temperature in wild birds, with context-specific ameliorating or suppressing effects on growth, depending on the thermal environment where the manipulation was performed (Dawson et al., 2005; Rodríguez and Barba, 2016a, b; Andreasson et al., 2018). Only one of these studies gives some insight into effects on thermoregulation: Andreasson et al. (2018) found that heated chicks maintained stable *T*_b_ throughout ontogeny despite *T*_a_ approaching 50°C, even at ages where control chicks were poikilothermic. Hence, at least part of the suppressive effects of postnatal *T*_a_ might reflect differential allocation of resources from growth to thermoregulation to avoid hypo- or hyperthermia. It is not known if any such changes remain until adulthood. However, the fact that warm *T*_a_ improved chick survival in a cold habitat (Dawson et al., 2005) and long-term survival in an intermediate thermal environment (Andreasson et al., 2018), but negatively affected survival in a hot and dry climate (Rodríguez et al., 2016), suggests this is a topic worthy of future investigation. In this context, it is interesting to note that in altricial birds (like those in the studies above), the HTPA axis matures during the first week after hatching (Debonne et al., 2008), which suggests that thermal sensivity may be greater postnatally than in precocical species.

## Future Directions

**FIGURE 2:**
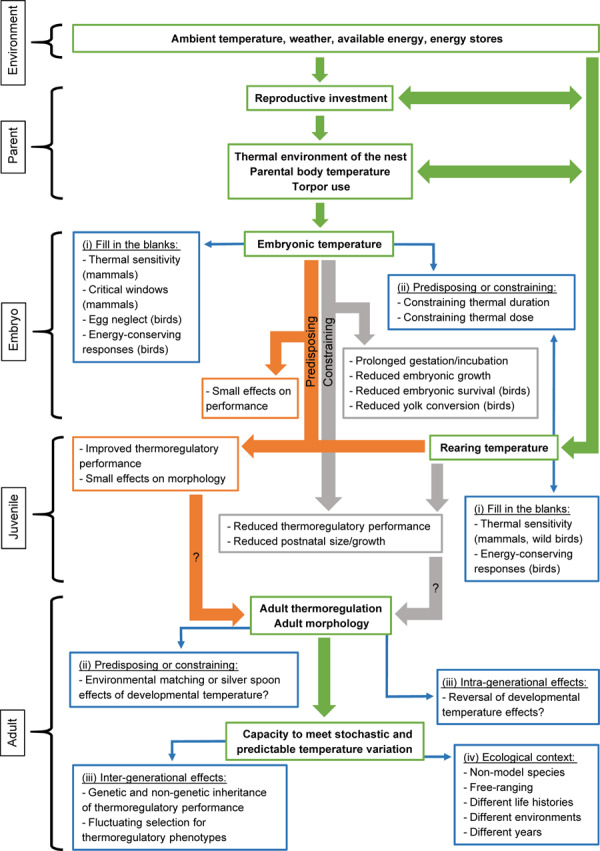
A putative flow path linking breeeding environment, parental investment, developmental and rearing temperatures, and thermal performance in adulthood. Main connections outlining how environmental and intrisic conditions experienced by parents during the breeding season can affect reproductive investment and resultant embryonic and postnatal thermal environments are shown using green arrows, with relevant descriptors in bold font within the green boxes. It is assumed that early life temperature can be either predisposing or constraining for subsequent thermoregulatory performance. These developmental trajectories are shown by orange and gray arrows, respectively, with relevant effects summarized within the orange and gray boxes. Paths where data are scarce, or even lacking, are traced using thin blue arrows, with key knowledge gaps listed within the blue boxes. For simplicity, these are referred to by the Roman numerals in the section “Future Directions.”

There appears to be broad synergies between studies in birds and mammals, despite variation in timing, duration, and severity of thermal stressors. In birds, there is a bias toward studies of production species with unclear ecological relevance, a general lack of information on effects of postnatal temperature on thermoregulation, and poor understanding of when a thermal dose is constraining or ameliorating. Mammals are comparatively understudied in all these regards. Hence, it is clear that more studies are needed to address how developmental temperature affects the ontogeny of thermoregulation and how this, in turn, impacts thermal physiology of adults. Below we outline some directions to further our knowledge of these matters (Figure 2):

– *(i) Fill in the blanks:* For example, there are few studies of thermal sensitivity of mammalian embryonic development and its short- and long-term consequences, despite widespread occurrence of heterothermy during pregnancy. In birds, there are no studies of how developmental temperature affects energy-conserving strategies, despite widespread heterothermy in this phylum (McKechnie and Lovegrove, 2002), and it is unknown how chick thermoregulation is affected by egg neglect.
– *(ii) Predisposing or constraining:* Increasingly stochastic climate suggests increased likelihood that an animal will develop during extreme weather, or that it will experience such events sometime during its lifetime. A key challenge is therefore to address if, how, and why, physiological changes that manifest during development affect performance when the juvenile and adult environments are mismatched. While subtle, short-duration, variation in developmental temperature can improve thermoregulatory performance in the same environment later in life, there are switch points where early-life temperature constrains subsequent temperature tolerance (e.g., Costantini et al., 2012). We need to understand better when a thermal dose transitions from predisposing to constraining, the phenotypic changes involved, and their epigenetic underpinnings (e.g., Vinoth et al., 2018; Wang et al., 2019). In this context, there is also a need for studies across life histories. For example, is environmental matching as relevant in a trans-continental migrant compared to a year-round resident (cf. Yin et al., 2019)?
– *(iii) Intra- and intergenerational effects:* There is a need to increase our understanding of the extent to which the thermo-physiological effects of developmental temperature remain over a lifetime, especially in wild models and mammals. To understand the evolution of responses, studies should address if traits that are differentially expressed in different developmental temperatures are heritable (cf. Rønning et al., 2007; Versteegh et al., 2008; Nilsson et al., 2009).
– *(iv) Broader ecological context:* Studies of physiological effects have used captive models, but fitness costs have been documented in the wild with little information on physiological mediators. We need to apply theory derived from captive models to wild animals that live under fluctuating *T*_a_ in a range of habitats, to better understand the eco-evolutionary dynamics of developmental thermal sensitivity.

## Conclusion

It is clear that mammals and birds are sensitive to fluctuating developmental temperature in broadly similar ways, and that changes brought about by the early thermal environment sometimes may permanently modify the phenotype. To this end, effects of temperature resemble those of other environmental factors during development (Costantini et al., 2010). Some studies, particularly in poultry, adhere to the environmental matching hypothesis (Figure 1A), showing that thermal acclimation in early life (*via* well-timed, brief, thermal manipulation) improves the capacity to meet matched stimuli in adulthood. However, wild and captive studies where the thermal challenge has been continuous (and the ecological relevance greater) adheres more closely to the silver spoon hypothesis (Figure 1B). That is, sustained deviation from the thermal environment to which the population is adapted seems to constrain phenotypic quality. However, we caution against general conclusions in this regard, because many key studies are yet to be performed (above and Figure 2), particularly in wild systems. Furthering our knowledge on how early life thermal conditions shape thermoregulatory phenotypes is more pressing now than ever when climate change increasingly exposes animals to extreme weather (IPCC, 2013) with potentially severe consequences (McKechnie and Wolf, 2010; Conradie et al., 2019; Riddell et al., 2019). Proper understanding of the ontogeny and acclimatization capacity of, and selection for, temperature tolerance is, thus, key to predicting how individuals and populations will respond to such challenges (cf. Stillman, 2003, 2019; Burggren, 2018). We hope that this review will inspire others to collect the data needed for better understanding of these effects.

## Author Contributions

AN and SG together developed the concepts of this mini-review, drafted its outline, and revised the manuscript. AN wrote the full version and produced the figures.

## Conflict of Interest

The authors declare that the research was conducted in the absence of any commercial or financial relationships that could be construed as a potential conflict of interest.
